# Phylogenetic Analysis Guides Transporter Protein Deorphanization: A Case Study of the SLC25 Family of Mitochondrial Metabolite Transporters

**DOI:** 10.3390/biom13091314

**Published:** 2023-08-28

**Authors:** Katie L. Byrne, Richard V. Szeligowski, Hongying Shen

**Affiliations:** 1 Cellular and Molecular Physiology Department, Yale School of Medicine, New Haven, CT 06510, USA; 2 Systems Biology Institute, Yale West Campus, West Haven, CT 06516, USA; 3 Yale College, New Haven, CT 06511, USA

**Keywords:** phylogenetic analysis, SLC25, mitochondria, metabolite transport, deorphanization

## Abstract

Homology search and phylogenetic analysis have commonly been used to annotate gene function, although they are prone to error. We hypothesize that the power of homology search in functional annotation depends on the coupling of sequence variation to functional diversification, and we herein focus on the SoLute Carrier (SLC25) family of mitochondrial metabolite transporters to survey this coupling in a family-wide manner. The SLC25 family is the largest family of mitochondrial metabolite transporters in eukaryotes that translocate ligands of different chemical properties, ranging from nucleotides, amino acids, carboxylic acids and cofactors, presenting adequate experimentally validated functional diversification in ligand transport. Here, we combine phylogenetic analysis to profile SLC25 transporters across common eukaryotic model organisms, from *Saccharomyces cerevisiae*, *Caenorhabditis elegans*, *Drosophila melanogaster*, *Danio rerio*, to *Homo sapiens*, and assess their sequence adaptations to the transported ligands within individual subfamilies. Using several recently studied and poorly characterized SLC25 transporters, we discuss the potentials and limitations of phylogenetic analysis in guiding functional characterization.

## 1. Introduction

It is estimated that at least 20–30% of the coding proteins derived from any model organism’s genome have poorly characterized functions [1]. To predict their functions, in silico analysis, especially sequence homology, is frequently used for functional annotation based on their characterized homologous proteins. However, this strategy is prone to false predictions due to functional diversification in protein evolution [2]. We therefore reasoned that the power of homology search in predicting function depends on the coupling of sequence variation to functional diversification, which can be evaluated by performing a phylogenetic analysis of homologs that span sufficient experimentally validated functional diversity.

In the context of cellular metabolism, specifically for metabolic enzymes and metabolite transporters of poorly characterized function, we consider functional prediction, the “deorphanization” effort, as predicting the ligands specifically binding at the evolutionarily conserved, putative catalytic pocket. This binding ligand can be a translocating ligand for a transporter, a substrate or product of a metabolic enzyme, or a regulatory molecule bound at the same pocket of these pseudogenized enzymes or transporters that gain additional moonlighting functions [3]. To test whether phylogenetic analysis is sufficient for guiding deorphanization and predicting putative ligands, we chose the mitochondrial SLC25 metabolite transporter family as the model family. 

The SLC25 family is the largest protein family responsible for translocating metabolites across the mitochondrial inner membrane, a process that critically controls all aspects of mitochondrial physiology [4,5,6,7,8]. The human genome encodes 53 SLC25 genes, including four adenine nucleotide carriers (ADP/ATP carriers) that exchange ADP^3−^ for ATP^4−^ to support cellular bioenergetics and UCP1, which is exclusively expressed in brown adipose tissue and dissipates the proton motive force to generate heat. Despite their critical roles, at least one-third of human SLC25 transporters remain poorly characterized, leaving ample space for deorphanization efforts. Meanwhile, decades of elegant experimental studies have provided functional and mechanistic insights, presenting an ideal case for evaluating the coupling of sequence variation and functional diversification for proteins within the SLC25 family. 

The SLC25 family is a prime candidate for our investigation into phylogenetic-analysis-guided studies for many reasons. First, the family is extraordinarily functionally diverse, being the largest protein family (53 in humans) that translocate a variety of chemically distinct metabolite ligands across the inner mitochondrial membrane, ranging from nucleotides to amino acids, carboxylic acids, inorganic ions, and different cofactors to protons with high specificity

Second, structure–function analysis, leveraging elegant primary sequence interrogation [9,10], atomic structures of different state conformations locked by pharmacological inhibitors [11,12] and site-directed mutagenesis screens [13,14], has confidently pinpointed the ligand binding residues of several characterized proteins in this family. This allows for an evaluation of coupling sequence adaptation to the transporting ligands. The transmembrane region of each SLC25 transporter is a structural and functional monomer of only approximately 300 amino acids with six transmembrane α-helices (threefold pseudo-symmetrical repeats of the two transmembrane helices called the “mito_carr” and “Solcar” domains) surrounding the central cavity. The ADP/ATP carrier crystal structures in both cytoplasmic-open (c-state) and matrix-open states (m-state), locked by two inhibitors bound at the ligand binding site, carboxyatractyloside and bongkrekic acid, respectively, highlight a conserved alternating-access transport mechanism that is triggered by a conformational change that occurs upon ligand binding at the central cavity [8]. Therefore, it is reasonable to speculate that the residues involved in ligand recognition and translocation would evolve and adapt for specific ligands that might be reflected by phylogenetic analysis.

Third, and most remarkably, the SLC25 family is highly evolutionarily conserved yet functionally diverse in eukaryotes. This family is present in all eukaryotic species (no bacterial and archaeal homologs have been identified so far), with only a few exceptions in protist species that have significantly reduced mitochondrial function, *Giardia lamblia* and *Encephalitozoon cuniculi* [15], or that have completely lost mitochondria, e.g., *Monocercomonoides* sp. [16]. We postulate that the SLC25 family might have massively expanded even before the last eukaryotic common ancestor and diversified into distinct transporters with different ligand specificities, subfamilies or orthologous groups. This is because (i) any genome that contains the SLC25 family is found to encode multiple SLC25 family genes, for instance, the human genome encodes 53, and even the distantly diverged apicomplexans contain about a dozen; (ii) in many reported cases [17,18,19], the corresponding mammalian ortholog can rescue the yeast SLC25 mutant phenotypes, suggesting human-to-yeast conservation; and (iii) previous phylogenetic analysis of SLC25 transporters within human and yeast genomes [20,21] already suggested that their protein sequences are clustered by function, specifically ligand specificity [21].

Here, to evaluate the sequence adaption for transporting ligands within the SLC25 family, we performed a phylogenetic analysis of SLC25 transporters from humans and commonly studied model organisms and annotated the tree based on the chemistry of the experimentally validated transporting ligands. We observed that for majority of the transporters, there exists a clustering of orthologous transporters across different species that transport the same ligand, suggesting an early divergence of these metabolite transporters as well as the coupling of sequence variation to ligand adaptation. They include ADP/ATP exchangers and transporters for CoA, carboxylates, glutathione, SAM and several amino acids, among others. In other cases, the sequence variation might be coupled to a conserved metabolite regulation mechanism or to other functional innovations, for instance, targeting different subcellular membranes, including outer mitochondrial membranes or peroxisomes. A lack of homology for several species-specific transporters is also noted. In summary, we explore the power of phylogenetic analysis in ligand prediction for the SLC25 transporter family, which might guide the “deorphanization” effort for some of the poorly characterized SLC25 transporters as well as provide an example for the exploration of other transporter families.

## 2. Materials and Methods

We chose the following five genomes, including commonly studied model organisms, to evaluate the SLC25 protein family: *Saccharomyces cerevisiae* (budding yeast, id: 4932), *Caenorhabditis elegans* (roundworm, id: 6239), *Drosophila melanogaster* (fruit fly, id: 7227), *Danio rerio* (zebrafish, id: 7955) and *Homo sapiens* (human, id: 9606). We reasoned that these organisms are diverse enough to represent different eukaryotic branches, and as model organisms, the sequences are more likely to be experimentally studied and thus suitable for our analysis.

SLC25 sequences from each genome were identified via a text-mining search in the UNIPROT database [22], using a combined “mito_carr” and “solcar” search into each organism via a taxonomy code (Data S1). We chose to carry out a text-mining search in UNIPROT over searching NCBI BLASTp/tBLASTn for the following reasons: first, the proteome annotations for these model organisms in UNIPROT are relatively complete, albeit with duplication and errors; and second, we found that “mito_carr” and “Solcar” domain predictions are of high accuracy. For instance, many SLC25 protein sequences from these model organisms are considered validated (“reviewed” in UNIPROT), including all 53 human SLC25s, all 35 yeast SLC25s and tens of worm, fly, and zebrafish SLC25s.

While validating the yeast sequences, we noticed that the Cmc1 protein from the S288c yeast strain (D6W196) was curated as a truncated form of the protein due to a frameshift in position 403 in Strain S288c. We therefore replaced this sequence with the full-length sequence of the same protein from the CG379 yeast strain (P0CI40).

Upon validating the yeast (35) and human (53) sequences, we then manually curated the SLC25 sequences in worm (49), fly (120) and zebrafish (111) using several rounds of multiple-sequence alignment (MUSCLE) [23,24] within each organism (Data S2). We (1) included all UNIPROT-“reviewed” sequences; (2) eliminated obvious duplicated sequences if the sequence identity <0.005; and (3) removed obvious truncated sequences (usually <200 amino acids in length). We chose 200 amino acids as a relatively loose cutoff to accommodate SLC25 proteins that might have truncated hydrophilic loops. However, all validated SLC25 protein sequences we curated are of approximately 300 amino acids or longer if they contain additional protein domains.

In summary, we included in our analysis 53 human, 35 yeast, 35 worm, 48 fly and 57 zebrafish SLC25 protein sequences. We acknowledge that our curation might contain errors for the worm, fly and zebrafish sequences, especially for paralogs within the same subfamily. First, for several subfamilies that are known to contain multiple paralogous genes, for instance, ADP/ATP exchangers and CMCs, multiple sequences might have identity values <0.01 but would either map to different chromosomal regions in tBLASTn or the mapping was of low confidence; therefore, the number of paralogs might be incorrect. Second, the zebrafish genome is known to have local genome duplication, so paralogs might be missing in our analysis. Regardless of the exact number of sequences, our analysis should sufficiently represent the complete functional diversity of the family.

We then manually trimmed all protein sequences to the regions that are predicted to encompass the three “Solcar” domains: the core transporter regions. For instance, the predicted EF-hand domains in CMCs and Aralars were removed. We then included the human MCU, another inner membrane channel, as the outgroup.

We aligned the protein sequences using MAFFT v7.505 [25] and determined the best substitution model (LG + F + G4) via ModelFinder [26]. We then used this model to construct a phylogenetic tree, using IQ-Tree v2.1.2. [27] with branch support values obtained via ultrafast bootstrapping from 10,000 replicates [28]. iTOL [29] was used to visualize and annotate the resulting tree, which we rooted using the human protein MCU as the outgroup.

## 3. Results

To perform the phylogenetic analysis, we collected amino acid sequences via a text-mining search (using “mito_carr” and “Solcar” in the UNIPROT database) against each model organism using their taxonomy codes. These organisms included *Homo sapiens* (human), *Saccharomyces cerevisiae* (yeast), *Caenorhabditis elegans* (worm), *Drosophila melanogaster* (fly) and *Danio rerio* (zebrafish). We chose these model organisms because they are evolutionarily diverse and are likely to be experimentally studied and annotated. Upon manual curation (see method), we finalized the sequence entry, which included 53 human sequences, 57 zebrafish sequences, 48 fly sequences, 35 worm sequences and 35 sequences. The large numbers of SLC25 transporters in all model organisms are consistent with a putative massive gene family expansion prior to the time when these eukaryotic lineages diverged. We then performed a multiple sequence analysis, using a nonhomologous mitochondrial calcium uniporter sequence in human (MCU) as the outgroup, and constructed the phylogenetic tree with bootstrap values to highlight clade confidence (a bootstrap value > 70 denotes high confidence).

**Figure 1:**
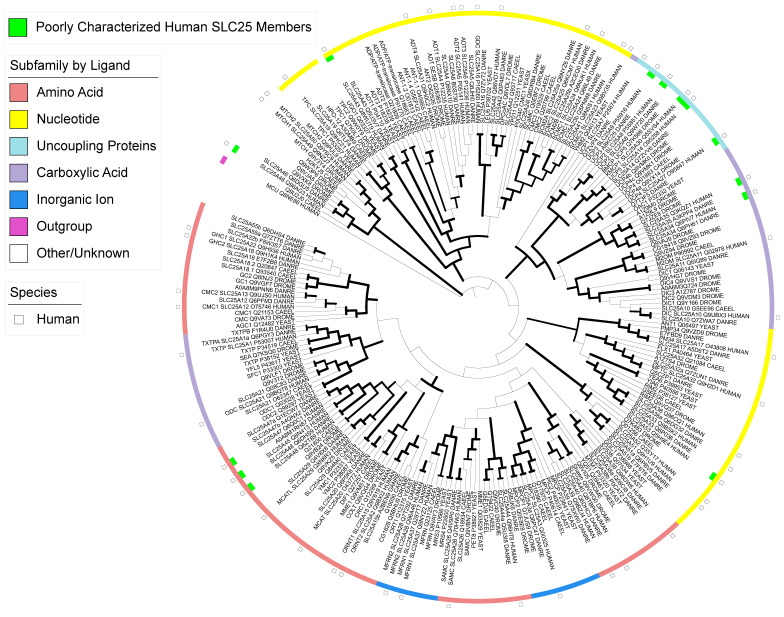
Phylogenetic analysis of SLC25 mitochondrial transporter family members. All human (human), *Danio rerio* (DANRE), *Caenorhabditis elegans* (CAEEL), *Drosophila melanogaster* (DROME), and *Saccharomyces cerevisiae* (YEAST, or YEASX for CMC1, see the Materials and Methods) SLC25 family transporters were aligned, built into a tree, then color-annotated by the transporting ligands. Bold lines represent bootstrap values > 70.

We then sought to functionally annotate the tree by color-coding major clades based on the chemistry of the transporting ligands identified for the known transporters in the clade. Specifically, the subfamily is manually annotated by ligand based on the biochemical activity of the well-characterized representative yeast or human SLC25 proteins in each clade and then expanded to the other SLC25 proteins in the clade with bootstrapping values > 90 (Figure 1 and Figure S1). Indeed, the majority of the well-characterized paralogs and orthologs across all model organisms translocating the same ligand are well-clustered with extremely high levels of confidence (bootstrapping values > 90), followed by homologs translocating ligands with similar chemical structures. For instance, the well-characterized SLC25 ADP/ATP exchanger (also named AACs, ANTs, ANCs and ADTs) that include four human transporters (SLC25A4, SLC25A5, SLC25A6 and SLC25A31) and their orthologs are all clustered together and are within the nucleotide transporter clade. Because several recent reviews have already summarized the characterized SLC25 transporters [21,30,31], here, we will highlight a few recently identified transporters and transporters of poorly characterized functions or of uncertainty in the human genome, with the goal of guiding future functional characterizations.

### 3.1. Recently Characterized Transporters

- Glutathione for SLC25A39 and SLC25A40

SLC25A39 was recently identified as the evolutionarily conserved, putative transporter for the major cellular antioxidant glutathione, the tripeptide in which the γ-peptide bond preserves the amino acid chemical group of the glutamate [32,33]. The phylogenetic tree supports SLC25A40 as the human paralog [32].

- BCAAs for SLC25A44

The recently identified branched-chain amino acid (BCAA) transporter SLC25A44 [34] contains orthologs in animals (fly, worm and zebrafish) but no obvious orthologs in yeast.

Both the SLC25A39 and SLC25A44 clades are nested within a cluster of transporters (despite having low homology with the other groups) in which the majority translocate amino acids, supporting their putative identification.

- NAD+ for SLC25A51 and SLC25A52

Recently identified putative human NAD+ transporters, SLC25A51 [35,36,37] and SLC25A52 [35], cluster within the same clade. This clade also contains orthologous genes in all animal model organisms but exhibits little homology with the yeast NAD transporters Ndt1/Yia6 and Ndt2/Yea6 [38]. It would be intriguing if fungi and animals independently evolved their ability to transport NAD cofactors, as well as their dependence on NAD-mediated OXPHOS to support mitochondrial bioenergetics. A homologous human transporter, SLC25A53, remains an orphan. This clade is adjacent to the majority of the nucleotide transporter clade, but a lack of high homology (bootstrap values < 70) might suggest that a homology search is not sufficient for guiding ligand characterization.

### 3.2. Transporters of Uncertainty

- SLC25A32

The evolutionarily conserved SLC25A32 subfamily has been proposed to transport both dinucleotide cofactor FAD and tetrahydrofolate (THF) cofactors supporting a mitochondrial one-carbon metabolism. While in vitro [39], mouse genetics [40] and cellular characterizations [41,42,43] supported its role in THF cofactor transport and one-carbon metabolism, this SLC25A32 clade’s homology to other nucleotide transporters might also support FAD transport [44,45,46].

- SLC25A38

The previously proposed putative glycine transporter SLC25A38 [47,48] was found to contain orthologs in zebrafish, yeast and human. This clade was recently proposed to also transport isopentenyl pyrophosphate (IPP), which is involved in CoQ biosynthesis (yeast ortholog Hem25, a fungi-specific function [49]), and to regulate other metabolite transport. The SLC25A38 clade appears to be within a majority amino-acid-transporting group.

### 3.3. Poorly Characterized Transporters

- Peroxisomal SLC25A17

The only non-mitochondrial SLC25 protein, peroxisomal SLC25A17 [50], which is present in human, fruit fly, and zebrafish, does not exhibit homology of high confidence with other SLC25 transporters. Its clustering with the yeast peroxisomal nucleotide transporter Ant1 [51] cannot be validated via a bidirectional best hit (BBH) search. This lack of obvious homology might suggest an early divergence with potentially broader substrate specificity [50] and a situation in which sequence variation might not be coupled to ligand specificity but to subcellular targeting.

- SLC25A34 and SLC25A35

The distinct SLC25A34 and SLC25A35 subfamily of completely unknown function contains human, fly, zebrafish and yeast orthologs, such as yeast Oac1 [52], but does not have any worm orthologs. The clade exhibits homology with the clade of dicarboxylate transporters SLC25A10 and SLC25A11 (bootstrap = 59), followed by homology with the UnCoupling Protein subfamily UCP1-5 (SLC25A7, A8, A9, A14 and A30) (bootstrap = 95), in which dicarboxylate transport activities have been proposed for several UCPs [53,54,55]. Such insights might guide the characterization of these proteins.

- SLC25A43

The vertebrate-specific SLC25A43 (only in human and zebrafish) of completely unknown function sparked interest because the clade is nested within a majority nucleotide group with high confidence (bootstrap = 86). The clade’s high homology to ADP/ATP carriers (SLC25A4, A5, A6 and A31), CoA transporters (A16 and A42) and the nucleotide-importer SCAMC subfamily, followed by thiamine pyrophosphate transporters, might guide the deorphanization of SLC25A43.

Despite a close clustering of high confidence, we cannot validate the homology via BBH search between SLC25A43 and the yeast Ugo1, an outer mitochondrial membrane SLC25 protein that is important for mitochondrial fusion with unknown ligand significance [56]. We therefore suspect that this clustering might be an artifact due to long-branch attraction of unknown causes [57], and the SLC25A43 clade might be a vertebrate-specific innovation.

- SLC25A45, SLC25A47 and SLC25A48

The homologous SLC25A45, SLC25A47 and SLC25A48 are three poorly characterized transporters that are clustered within a distinct clade within the amino acid transporters, separated (bootstrap = 83) but homologous (bootstrap = 78) with the putative basic amino acid transporter SLC25A29, followed by a homology with the carnitine transporter SLC25A20 and the ornithine transporters SLC25A15 and SLC25A2 (bootstrap = 100). Recent loss-of-function studies in mouse livers suggest that deleting the liver-specific [58,59,60] *Slc25a47* leads to a reduction in respiration [58], an increase in the mitochondrial stress response [58,60] and an impaired lipid metabolism [59]. While the exact endogenous ligand is yet to be identified, our phylogenetic analysis does not support a nucleotide substrate using this family-wide conserved ligand recognition mechanism.

- Outer mitochondrial membrane SLC25A46, MTCH1 and MTCH2

The three human outer mitochondrial membrane SLC25 proteins, SLC25A46, MTCH1 and MTCH2, are homologous with high levels of confidence with other animal sequences in worm, fly and zebrafish. A lack of homology with the yeast outer mitochondrial membrane protein Ugo1 might suggest two independent evolutionary innovations to utilize the inner membrane SLC25 protein scaffold for new outer membrane function. The recently proposed novel role of MTCH2 as an outer mitochondrial membrane insertase [61] is intriguing, and future studies are required to bridge this biochemical activity with the previously proposed roles of MTCH2 in membrane fusion [62,63] and apoptosis [64] in yeast and in animals. The significance of potential metabolite binding at the family-wide conserved ligand binding pocket is yet to be explored.

## 4. Discussion

Here, we performed a phylogenetic analysis of SLC25 family transporters from common model organisms to evaluate the power of homology search in guiding ligand characterization. Our analysis exhibited an extremely high degree of homology between SLC25 transporters across evolution that transport the same ligand (bootstrapping value > 90), consistent with an early functional divergence of SLC25 proteins. Consistent with previous phylogenetic analyses [20,21], we also observed a high degree of homology between SLC25 proteins transporting ligands with similar chemistries, e.g., nucleotides, amino acids and carboxylates (bootstrapping value usually >50), supporting the coupling of sequence variation to functional divergence. The ability of the homology search to guide ligand prediction is promising for several remaining “orphan” SLC25 transporters and is discussed case-by-base.

While several phylogenetic analyses have been performed on automatically collected SLC25 sequences across a broad range of eukaryotic taxa [65,66,67], our analysis, which focused on a few model organisms, provides unique insights to guide future functional annotation. Because the model organism proteomes are well curated, we were able to carefully exhaust all SLC25s in each species and more importantly, immediately distinguish the evolutionarily conserved SLC25s from the SLC25s that might be due to recent innovation in vertebrates or in mammals. Unsurprisingly, the biochemical activities of most evolutionarily conserved SLC25s have been characterized, and a majority of the poorly characterized transporters are only present in certain species, suggesting they are dispensable for central and conserved mitochondrial metabolism in animals—an important insight to interpret for any future research detecting their biochemical activities in the context of the human mitochondrial metabolism.

Our analysis should not be affected by the potentially incorrect number of SLC25 family proteins in each species because the error should only occur for species-specific paralogs for which we cannot distinguish whether certain transporter sequences have inaccurate annotations or are taxa-specific gene duplications. A more complete analysis might benefit from the inclusion of other model organisms, including the plant *Arabidopsis thaliana*, house mouse, *Mus musculus*, and western clawed frog, *Xenopus tropicalis* (often used for the study of ion channels). For recently innovated SLC25 proteins, a phylogenetic analysis focusing on sequenced vertebrate [68] or mammalian genomes [69] might inform the understanding of their adaptative evolution in human physiology.

Several limitations to utilizing homology search for ligand characterization are noted. First, singleton transporters with little homology with other transporters provide little guidance toward their characterization. Examples of this include the peroxisome SLC25A17 clade (discussed above) and many species-specific transporters, for instance, the fruit fly MME1 (Q9VM51, annotated as “Mitochondrial Magnesium Exporter 1”) [70] and the COLT of unknown function (Q9VQG4, annotated as “Congested-like trachea protein”) [71], both of which are within the amino acid transporter clade. Second, we highlighted that homology-based sequence variation cannot distinguish between adaptation to new ligands and adaptation to different subcellular organelle targeting or to other moonlighting functions. For instance, the homology among the UnCoupling Proteins (UCPs) might potentially suggest an adapted metabolite regulation mechanism instead of an adaptation to transported ligands—for example, the regulation of UCP1 by purine nucleotides—demonstrated via structure [72,73] and molecular simulation [74].

A phylogenetic analysis could potentially guide experimental ligand characterization efforts for certain SLC25 transporters as an orthologous strategy to in vitro proteoliposome-based transport assays, cell-based assays and in vivo studies. Other molecular evolution approaches could also provide exciting insights [66]. A comprehensive evaluation of how a homology search and phylogenetic analysis could guide deorphanization efforts probably requires a “dream world” scenario in which experimentally validated functional annotation is available for every Cluster of Orthologous Group (COG) in bacteria and in eukaryotes [75].

## Data Availability

Sequence and alignment data files are provided within the paper.

## Supplementary Materials

The following supporting information can be downloaded at: https://www.mdpi.com/article/10.3390/biom13091314/s1, Figure S1: Phylogenetic analysis of the SLC25 mitochondrial transporter family members with bootstrap values; Data S1: SLC25 transporter protein sequence file; Data S2: SLC25 transporter protein alignment file.
