# Supplementary material for: Phylogenetic Analysis Guides Transporter Protein Deorphanization: A Case Study of the SLC25 Family of Mitochondrial Metabolite Transporters
Source: Biomolecules. 2023 Aug 28;13(9):1314. doi: 10.3390/biom13091314 (PMC10526428; doi:10.3390/biom13091314)
Supplement: Supplementary file 1 [file biomolecules-13-01314-s001.zip › Figure S1.pdf]

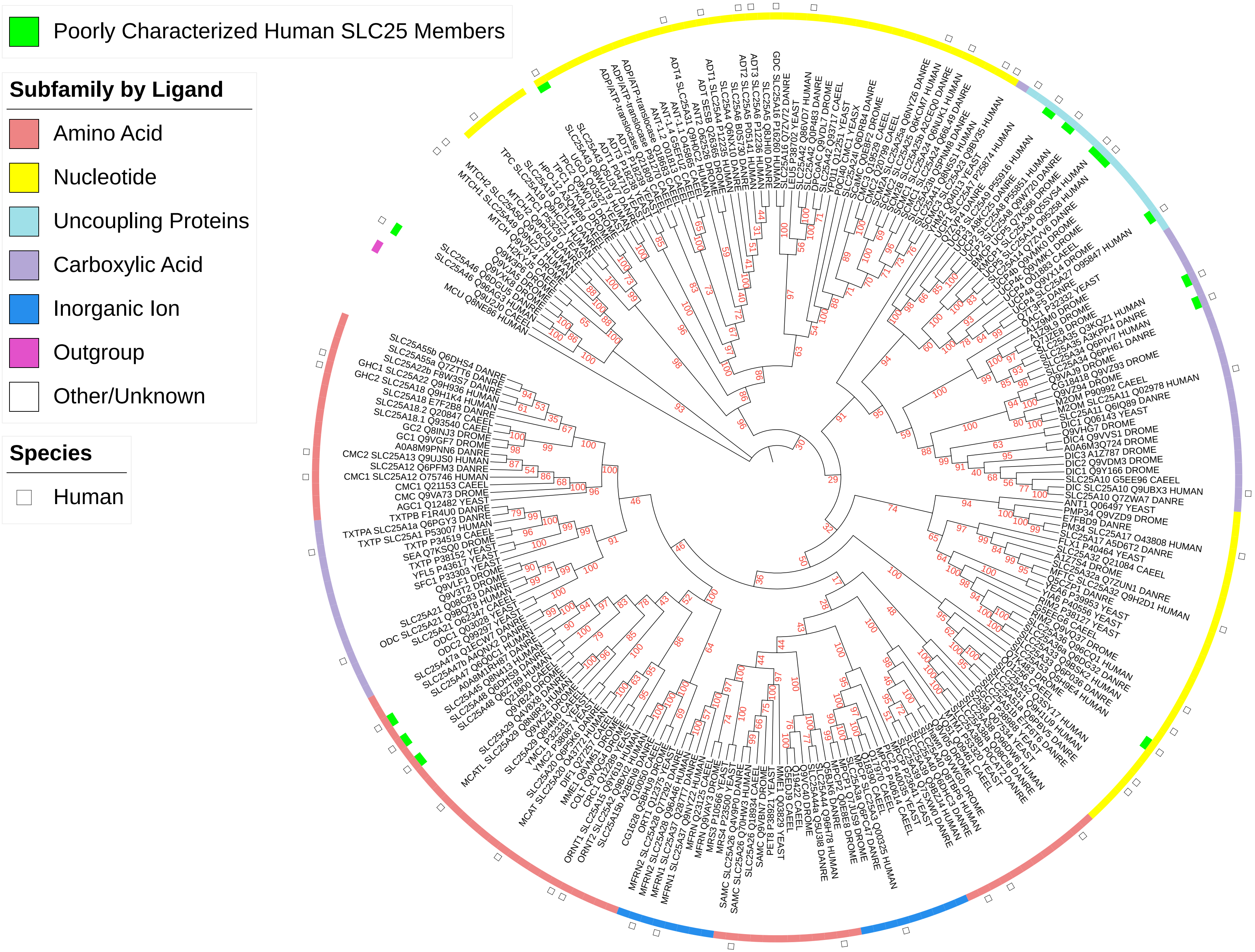

Figure S1. Phylogenetic analysis of the SLC25 mitochondrial transporter family members with bootstrap values.
